# Effect of Fermentation Using *Lactobacillus plantarum* A6 on the Physicochemical and Functional Properties of Precooked *Sorghum bicolor* and *Voandzeia subterranea* Blended Flour

**DOI:** 10.1155/2020/9234083

**Published:** 2020-12-09

**Authors:** Thierry Marcel Beumo Ntsamo, Bouba Adji Mohammadou, Alphonse Tegang Sokamte, Nicolas Yanou Njintang, Leopold Ngoune Tatsadjieu

**Affiliations:** ^1^National School of Agro-Industrial Sciences, Department of Food Science and Nutrition, University of Ngaoundere, P.O. Box 455, Ngaoundere, Cameroon; ^2^University Institute of Technology, Department of Food Engineering and Quality Control, University of Ngaoundere, P.O. Box 454 Ngaoundere, Cameroon; ^3^Faculty of Sciences, Department of Biological Sciences, University of Ngaoundere, P.O. Box 454, Ngaoundere, Cameroon

## Abstract

The present study is aimed at producing *Sorghum bicolor* and *Voandzeia subterranea* complementary instant flour. The precooked sorghum and Bambara groundnut flours were mixed at different proportions (0 : 100, 25 : 75, 50 : 50, 75 : 25, and 100 : 0), hydrated with sterile distilled water (1 : 3, w:v), and fermented for 72 h/37°C using *Lactobacillus plantarum* A6 at 10^5^ CFU/g. During the fermentation, samples were withdrawn for pH, titrable acidity, and microbial analyses. After fermentation, color, particle size, water absorption capacity (WAC), solubility index, least gelling concentration (LGC), and physicochemical and pasting properties were determined. The results showed that the fermentation significantly (*p* ≤ 0.05) decreased WAC, LGC, peak viscosity, final viscosity, breakdown, and pH, but increased the solubility index and titrable acidity of flours. The protein and carbohydrates contents as well as the color, particle size, and the setback after the cooking of the flour were not significantly affected by the fermentation. The flours ratio and fermentation also significantly decreased the total polyphenols, tannins, and phytate content of the samples. The fermented blended flour containing 25% precooked sorghum flour and 75% roasted Bambara flour (SVFP25) is a promising alternative as instant flour used for young children's nutrition.

## 1. Introduction

More than 200 million children under five around the world are malnourished [1] and living mainly in developing countries. This is mainly due to the poor dietary practices and low nutritional quality of food used for infants' and children's nutrition. Indeed, to ensure adequate infant feeding, exclusive breastfeeding from birth to 6 months as well as adequate complementary feeding introduced from 6 months up to 24 months or more with continued breastfeeding is recommended [2]. Complementary food is food used for infant feeding along with breast milk feeding when breast milk alone is no longer sufficient to meet all nutritional requirements [3]. It should have about 92-95% of dry matter, 68% of carbohydrates, 15% of proteins, 8% of lipids, 3.2% of fibers, 2.9% of ash, and a caloric value of about 400 kcal/100 g [4]. In Africa, starchy flours from tubers or cereals are generally used as raw material by local populations to prepare gruels used as a complementary food for infant nutrition. However, these gruels due to their high viscosity, low energy density (20-40 kcal/100 mL), and nutritional quality [5] are inappropriate for the young child feeding. The high viscosity of these gruels is due to the gelatinization of the native starch during cooking.

To reduce this viscosity and increase the energy density of the gruels, the partial hydrolysis of starch in flour before, during, and after the production of flour by thermal [6] or enzymatic modification (germination, use of amylase, fermentation with amylolytic microorganisms) have been proposed [7, 8]. Beyond the starch hydrolysis, the fermentation of starchy products using selected microorganisms like lactic acid bacteria (LAB) improves the nutritional and sensory values, as well as microbiological safety of the final products [9, 10]. LAB are able to ferment different types of starchy products such as corn, potatoes, and millet [11, 12]. Judiciously selected, a pure strain of amylolytic lactic acid bacteria use for fermentation could allow the production of fluid gruel with high energy density. However, it has been shown that additional treatments such as cooking and roasting can be applied prior to the fermentation of starchy raw materials in order to obtain a fluid slurry with a much-improved energy density, as well as better nutritional quality. For instance, Haydersah et al. [13] show that when *Lactobacillus plantarum* is used to ferment the precooked starch, there is a greater decrease in the viscosity compared to native starch of the resulting porridge, and consequently a better increase in its energy density. Furthermore, the use of leguminous seeds during this flour preparation can allow improving its nutritional value, mainly the protein content and protein quality.

Sorgum (*Sorghum bicolor* L. Moench) and Bambara groundnut (*Voandzeia subterranea* L. Thouars) are two indigenous crops of tropical Africa widely cultivated for their seeds used as foodstuff. The annual production of sorghum in Africa is estimated at around 20 million tons, and it has the advantage of tolerating adverse conditions such as hot, dry, wet, and water-logged conditions [14]. Its grains contain mainly carbohydrates (77%), proteins (11%), and lipids (3%) [15]. Bambara groundnut is the third important leguminous after groundnut and cowpea in Africa [16]. In Cameroon, its production was estimated in 2013 at 36,639 tons [17]. It can grow on low fertile soils of dry regions and tolerate fairly acidic soils [18]. Its seeds have high nutritional value with high protein content (19-28%), are highly caloric (about 387 kcal/100 g), and are rich in several minerals (magnesium, calcium, iron, manganese, sodium, potassium) and vitamins (thiamine, riboflavin, niacin, pantothenic, ascorbic acid, pyrodoxine, alpha-tocopherol, and vitamin K) [19–21]. Because of its nutritional and technological potential, the Bambara groundnut has been found to be a good leguminous crop to enrich the protein content of a maize-based pudding (abari) [22]. However, to the best of our knowledge, and despite the nutritional and technological potential of sorghum and Bambara seeds, the valorization of their seeds as the main raw material in the production of infant food flour has not been the subject of any published scientific study.

The aim of this study was to evaluate the effect of combining heat treatments and fermentation with *Lactobacillus plantarum* A6 strain on the quality of an instant flour food made with sorghum and Bambara groundnut flours as raw materials.

## 2. Materials and Methods

### 2.1. Raw Material

The dry sorghum grains of *madjeru* variety and dry white Bambara groundnut were purchased from local markets in Ngaoundere (Adamaoua region, Cameroon) in June 2018.

### 2.2. Stater

*Lactobacillus plantarum* A6 strain used for the fermentation was provided by the Microbiology Laboratory of CIRAD Montpellier, France.

### 2.3. Production of Precooked Flours

**Figure 1:**
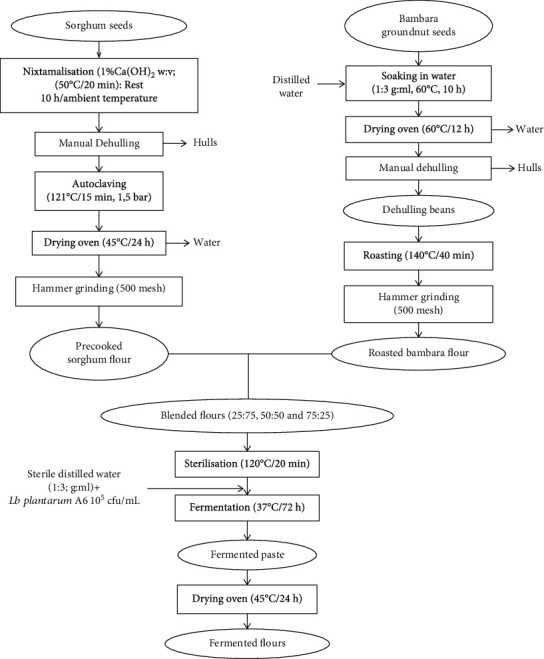
Flow diagram for the production of sorghum and Bambara groundnut precooked and fermented flours.

The precooked flours were produced as shown in Figure 1. Sorghum grains were sorted manually to remove stones and other impurities. The clean seeds were then soaked (1 : 3, w:v) in a solution of Ca(OH)_2_ (1.5 g/L) at 50°C for 20 min in the water-batch, then removed and maintained at room temperature (25 ± 2°C) for 10 h and manually dehulled. The dehulled grains were then precooked at 121°C/15 min in the autoclave (Caudice Civab Autosa 76, Roma, Italy), drained, and cooled at room temperature on stainless trays and dried at 45°C for 24 h in a ventilated electric dryer (Riviera and Bar, France). The dried cooked grains were ground in a hammer mill (Culatti, polymix, France) fitted with a 500 *μ*m pore size sieve.

Bambara seeds were manually cleaned thoroughly, and foreign materials as well as broken and immature seeds were removed. The clean seeds were soaked in distilled water (1 : 3, w : v) at 60°C for 8 h, drained on stainless trays, and dried at 60°C for 12 h in the oven. The dried seeds were dehulled by abrasion using hand millstone and winnowing, roasted in a laboratory roaster (Torre Picenardi, PANACEA 430, Italy) at 140°C for 30 min, cooled at room temperature, and milled with a hammer mill (Culatti, polymix, France) fitted with a 500 *μ*m pore size sieve. The different flours produced were sealed in high-density polyethylene bags and stored in a dry condition at laboratory temperature for further uses and analyses.

Mixtures of precooked sorghum and Bambara groundnut flours were formulated using different proportions 0 : 100, 25 : 75, 50 : 50, 75 : 25, and 100 : 0 (sorghum : Bambara groundnut, w : w).

### 2.4. Fermentation of Blended Flours

#### 2.4.1. Cultivation of *Lactobacillus plantarum* A6

The stock culture of *Lb. plantarum* A6 used was precultured in Man Rogosa Sharp (MRS: yeast extract: 5 g; meat extract: 5 g; peptone: 10 g; sodium acetate: 5 g; sodium citrate: 2 g; glucose: 20 g; potassium phosphate: 2 g; magnesium sulphate: 0.1 g; manganese sulphate: 0.05 g; tween: 1 mL) broth at 37°C for 24 h. After this period, the volume of the broth was completed to 1 L under aseptic conditions with sterile MRS broth, homogenized, and incubated at 37°C for 16 h. The resulting culture broth was centrifuged (Hereaus Biofuge primo R, Germany) at 6500 rpm at 4°C for 20 minutes. The bottom was collected, washed thrice with sterile saline water (8.5 g/L NaCl), and used to prepare inoculum.

#### 2.4.2. Inoculation and Fermentation

For fermentation, 500 g of the samples from each flour and mixture were introduced into 2500 mL glass bottle and autoclaved at 121°C for 15 min. After cooling at room temperature in aseptic conditions, each flour was hydrated using sterile distilled water (1 : 3, w : v). The slurry obtained was inoculated with 3 mL of suspension of *Lb. plantarum* A6 to achieve a final concentration of 10^5^ CFU/g, then homogenized aseptically with a sterile glass rod and incubated at 37°C for 72 h.

### 2.5. Microbial Count and Chemical Analysis during Fermentation

During fermentation, the samples were withdrawn at 0, 2, 4, 6, 8, 12, 16, 20, 32, 44, 56, and 72 h. Before drying, the pH, titrable acidity, and microbial count of the samples were determined. The pH of the sample was measured by introducing the glass electrode of the pH meter into the sample [23]. The titrable acidity was measured using the method described by AOAC [24]. Ten milliliters of the sample slurries was titrated using 0.1 M NaOH with phenolphthalein as an indicator. The amount of acid produced was expressed as a percentage of equivalent lactic acid. The *Lb. plantarum* A6 count of the fermented samples was determined according to the modified method described by Leclerc et al. [25]. One gram of the fermented sample was mixed with 9 mL of saline water (8.5 g/L of NaCl) and homogenized with a vortex. Serial decimal dilutions were performed and 0.1 mL of each dilution spread on MRS agar (yeast extract: 5 g; meat extract: 5 g; peptone: 10 g; sodium acetate: 5 g; sodium citrate: 2 g; glucose: 20 g; potassium phosphate: 2 g; magnesium sulphate: 0.1 g; manganese sulphate: 0.05 g; agar: 15 g; tween: 1 mL), incubated for 24 h at 37°C. Microbial loads were expressed as log10 colony-forming units (CFU) per gram of sample.

### 2.6. Chemical, Physical and Functional Analyses of Flour

The fermented doughs were spread in a thin layer on a stainless steel tray and dried in a ventilated oven at 45°C for 24 h, then ground in a hammer mill (Culatti, polymix, France), put in sterile bags in polyethylene, and stored at 4°C for further analysis. The analyses covered both fermented and unfermented flours.

#### 2.6.1. Determination of the Proximate Composition and Some Phytochemicals

The flours were analyzed for dry matter and ash content [24]. The lipid content was determined by Soxhlet extraction using hexane according to the method described by Bourely [26], total sugar content was analyzed according to the Dubois method [27], and total protein (*N* × 6.25) was analyzed by the Devani et al. [28] method after sulfuric acid digestion using AACC method [29]. The crude fiber content was determined according to the method described by Hassan and Umar [30]. The caloric value of the flours expressed in kcal/100 g DM was determined using the Atwater values for fat (9.0 kcal/g), protein (4.02 kcal/g), and carbohydrates (4.0 kcal/g) as described by Kumar and Sharma [31]. Phenolic compounds, tannins, and phytates were determined using the method described by Marigo [32], Vaintraub and Lapteva [33], and Phillips et al. [34], respectively.

#### 2.6.2. Physical and Functional Analyses of Flour

The colour parameters *L*^∗^, *a*^∗^, and *b*^∗^ (where *L*^∗^ is the lightness, *a*^∗^ the redness, and *b*^∗^ the yellowness) of the flours were measured using a tintometer (NH3R10, Nippon Denshoku Inc., Tokyo, Japan). A standard white plate (*L*^∗^ = 93.87, *a*^∗^ = 0.18, and *b*^∗^ = 2.72) was used for the calibration before measurement.

The average size and the particle size distribution were determined with laser light scattering apparatus (Mastersizer S 2000, Malvern Instruments Ltd., Malvern, UK). The raw data from the optical unit is processed by the Sizer Sv2.17 software (Malvern Instrument, Orsay) to calculate the particle size.

The water absorption capacity and the solubility index of the flours were determined according to the method described by Phillips et al. [34]. The bulk density and the least gelling concentration (LGC) were determined using the method described by Okezie and Bello [35] and Coffmann and Garcia [36], respectively.

The pasting properties of suspensions with 10% dry matter were measured using a viscograph (VAN, Newport Scientific, Narrabeen, Australia). These suspensions were dispersed by stirring at 960 rpm for 10 s then equilibrated at 50°C for 1 min, heated at 6°C/min to 95°C, maintained at 95°C for 5 min, and cooled from 95°C to 50°C at 6°C/min. During the analysis, the samples were continuously homogenized at 160 rpm. The pasting temperature (Pt), peak viscosity (PV), final viscosity (FV), hot paste viscosity (HPV), setback (SB), and breakdown (BD) were calculated from the pasting curve using the Thermocline v. 3.15 software (Newport Scientific).

### 2.7. Statistical Analysis

The effect of fermentation and the mixture proportion on the physicochemical and functional properties of flours were evaluated using the analysis of variance. The Duncan test was used to evaluate the differences between means. The statistical analysis was performed using Statgraphics Centurion XV software, whereas the Sigma plot 11 software was used to draw curves.

## 3. Results and Discussion

### 3.1. pH, Titrable Acidity, and Bacterial Growing Kinetics during Fermentation

**Figure 2:**
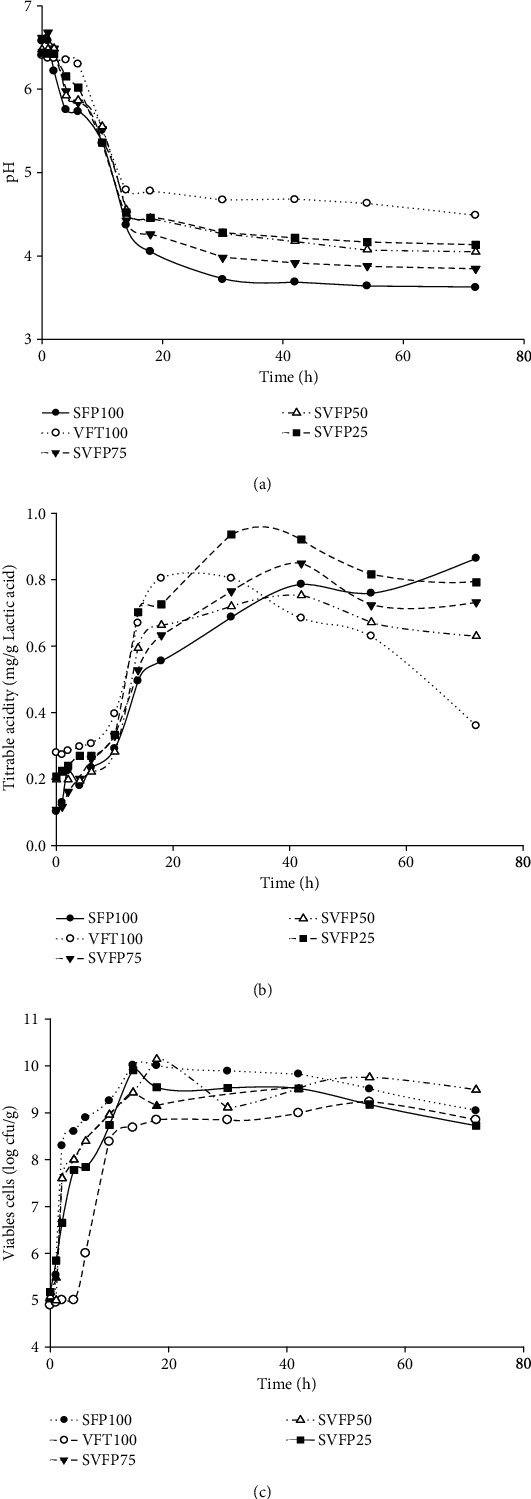
Evolution of pH (a), lactic acid (b), and *L. plantarum* A6 counts (c) during fermentation. SFP100: precooked and fermented sorghum flour; VFT100: roasted and fermented Bambara flour; SVFP25: fermented flour containing 25% of precooked sorghum flour and 75% roasted Bambara flour; SVFP50: fermented flour containing 50% precooked sorghum flour and 50% roasted Bambara flour; SVFP75: fermented flour containing 75% precooked sorghum flour + 25% roasted Bambara flour.

The effect of fermentation and flour proportion on pH, titrable acidity, and bacterial growth was evaluated, and the results are presented in Figure 2.

The evolution of pH and titrable acidity were significantly (*p* < 0.05) affected by the proportion of flour in the mixture. Indeed, the pH was higher with the content of Bambara groundnut flour in the mixture and varied from 4.05 for the sample with 25% of Bambara groundnut (SVFP75) to 4.25 for the sample with 75% of Bambara groundnut flour (SVFP25), and at the same time, there was a decrease of titrable acidity with the increase of Bambara flour in the mixture. This evolution is due to the fact that Bambara groundnut is higher in protein content and makes it and the blend less acid than sorghum flour. Nevertheless, the pH remained below 5. During the first sixteen hours of fermentation, the pH of the samples significantly decreased from 6.40 to 4.80 in roasted and fermented Bambara groundnut (VFT100) and from 6.57 to 4.37 in precooked and fermented sorghum (SPF100). After this period, the pH of the samples remained almost constant with slight decrease from 3.85 to 3.59 for the mixture containing only precooked sorghum flour (SPF100). The decrease in pH of the samples may be mainly due to the production of the organic acids, showing the capacity of *Lb. plantarum* A6 to ferment sugars in Bambara groundnut and sorghum flours as well as their mixture. Indeed, during the fermentation, the titrable acid measuring the total organic acid contents of the samples significantly (*p* < 0.05) increased from 0.11 mg/g lactic acid equivalent in the flour with 25% of sorghum (SVFP25) to 0.94 mg/g lactic acid equivalent after 40 h. This result is similar to those obtained by Worku and Sahu [37] in the fermentation of red beans.

Figure 2(c) shows the growth of *Lb. plantarum* A6 in the different samples. The bacterial load increased rapidly from 5 log CFU/g to 10 log CFU/g after 20 h of fermentation, then remained constant until the sixtieth hour of fermentation and decreased up to 8.4 log CFU/g and 8.5 log CFU/g for VFT100 and SVFP25, respectively, at the end of the fermentation. The fast growth of the *Lb. plantarum* A6 in the samples shows its good aptitude to growth on sorghum and Bambara groundnut flour pastes. Indeed, *Lb. plantarum* has been found in many local fermented starchy products as one of the main natural fermenting bacteria [38]. The ability of *L. plantarum* A6 to ferment leguminous products had also been reported by Pahane et al. [39]. Nguyen et al. [8] have also reported similar results during the fermentation of gelatinized rice/soybean slurries.

### 3.2. Proximate Composition of Blended Flours

Table 1 shows the proximal composition of the different flours. The dry matter of the flours varies during the fermentation and decreases from 95.6% to 94.53% for SVP25 and from 94.0% to 92.13% for SVP50. This result shows that fermentation decreases the dry matter of flour, except in the case of SVP75, for which the fermentation leads to an increase in dry matter from 93.13% to 94.53%. Fermentation would, therefore, cause changes in the chemical composition of flour. Moreover, dry matter also increases with an increase of Bambara groundnut proportion into the mixture from 93.13% for the precooked and blended flour with 25% of Bambara groundnut (SVP75) to 95.6% for the precooked and blended flour with 75% of Bambara groundnut (SVP25).

The ash content of flours ranged from 2.27 to 2.48 g/100 g DM in unfermented samples and 1.86 to 2.91 g/100 g DM in fermented blends (Table 1). In general, the ash content of the different flour was not significantly (*p* > 0.05) affected by the fermentation. These results are different to those reported by Oyarekua and Bankefa [40] who showed a decrease of ash content in cooked maize and walnut after fermentation as a result of usage of minerals by increased metabolic activities of inherent microorganisms. In the case of increasing the ash content of the SVP75 flour, similar results were also reported by Simwaka et al. [41] on the fermentation of pumpkin and amaranth blended flours at different proportions. However, the substitution of sorghum flour by Bambara groundnut flour mainly at 75% and 50% significantly (*p* < 0.05) decreased the ash content of the fermented blended flour and could be due to the low ash content of the Bambara groundnut precooked flour used (Table 1).

Fat content increased with the Bambara flour rate in the mixture and is due to its higher fat content compared to sorghum flour (Table 1). However, the fat content of the samples was not significantly (*p* ≤ 0.05) affected by fermentation. The fat content of SVP50 is 6.20 g/100 g DM and that of SVP75 is 5.14 ± 0.04 g/100 g DM. After fermentation of these flours, the fat content is 6.46 g/100 g DM and 5.45 g/100 g DM, respectively, for SVFP50 and SVFP75. Thus, there would be no significant variation in the lipid content after fermentation. However, there was a slight increase of 0.92 g/100 g DM on the flour blend containing 75% Bambara groundnut, ranging from 7.23 g/100 g DM for SVP25 to 8.15 g/100 g DM for SVFP25. This slight increase would be the result of the concentration of certain nutrients as a result of the reduction in sugar content since the fact that *Lb. plantarum* metabolizes mainly sugar during the fermentation. This result differs from that has been found by Chinenye et al. [42] who reported the decrease of fat content in pearl millet flour after fermentation. This difference could be explained by the type of microorganism used for fermentation. Among the different samples, the fermented flour with 25% of sorghum and 75% of Bambara groundnut (SVFP25) having 8% of fat is suitable for use in complementary weaning food [4].

Protein contents increased with fermentation and the substitution rate. It is significantly and positively correlated to Bambara groundnut content and lies between 12.10% in precooked sorghum and 19.98% in fermented flour containing 75% of Bambara groundnut (SVFP25). This is attributed to high levels of protein content in Bambara groundnut. There is a slight increase in the protein content during fermentation and can be due to the activity of *Lb. plantarum* A6 with the synthesis of new cells rich in protein while decreasing the carbohydrate content by fermentation. Ojokoh et al. [43] also reported improvement and protein content of food by fermentation.

The total sugar content of the samples ranged from 69.65% in SP to 60.35% in VT (Table 1). For the mixtures, the total sugar content decreased by up to 62.76% with the increase of the Bambara flour rate in the flour mixture (SVP25). This result could be explained by the lower total sugar content in the Bambara groundnut compared to that of sorghum flour. In general, the total sugar content of the fermented samples (60.66, 62.51, and 64.37%, respectively, for SVFP25, SVFP50, and SVFP75) is lower than that of unfermented flours (62.76, 64.88, and 67.58%, respectively, for SVP25, SVP50, and SVP75). The decrease in total sugar during fermentation is the fact of the utilization of sugars by *Lactobacillus plantarum* A6 for metabolic activities, mainly as an energy source [44]. The variation of fat, protein, and sugar content affects the caloric value of the samples (Table 1). The caloric values range from 363.04 ± 1.91 kcal/100 g DM in the unfermented sorghum flours (SP) to 400.61 ± 4.72 kcal/100 g DM in roasted Bambara flour (VT). Thus, the increase in the level of Bambara flour in the mixture significantly increased the caloric value of the sample. This is due to the higher caloric value of Bambara groundnut thanks to its high-fat content. There was a slight increase in the caloric value of the samples after fermentation as the fat and protein contents increased in these flour samples at the end fermentation process.

The crude fiber content of the samples was not significantly affected by the fermentation and varies from 1.76, 1.54, and 1.40% in unfermented blended flour (SVP75, SVP50, and SVP25, respectively) to 2.52, 2.23, and 2.09% in fermented blended flour (SVFP75, SVFP50, and SVFP25, respectively) (Table 1). Different results were reported [45] with a significant reduction of the crude fiber content of sesame and melon after natural fermentation, and this could be attributed to microbial strains used.

### 3.3. Antinutrient Composition of Blended Flours

The results of the analysis on the antinutrient composition of the different flours are shown in Table 2. The total polyphenol content of the different flours produced decreased during fermentation, lying between 117.02 mg/100 g DM in unfermented flour (SVP25) and 29.05 mg/100 g DM after fermentation in SVFP25. The reduction in total polyphenol compounds could be related to the activity of the polyphenol oxidase of the microflora used for the fermentation [46]. Similar results were reported by Noumo et al. [44] using *Lb. plantarum* A6 for the fermentation of Moringa leaf powder. The ratio of the flours in the mixture also significantly influenced the polyphenol content of the samples. Indeed, the increase in the proportion of Bambara also increased the total polyphenol content of the flour mixture.

The fermentation of the flours induced a decrease in their tannin content from 50 mg/100 g DM in SVP75 to 11 mg/100 g DM in the fermented flours. There was no significant difference between the fermented flours regardless of the proportions of the mixtures. The reduction of total tannins could be due to the tannase activity of *Lactobaccillus* [47].

The phytate contents of the various flour samples are shown in Table 1. Globally, the fermentation reduced by 70% the phytates content of the mixture of precooked sorghum flour (25%) and roasted Bambara flour (75%). Also, the reduction of the proportion of Bambara in the mixture showed the reduction of total phytates content in the blends. The reduction of phytate content during fermentation could be attributed to the ability of *Lb. plantarum* A6 to produce phytases [48]. Indeed, Noumo et al. [44] also reported the reduction of phytate contents in Moringa leaf powder during fermentation by *Lb. plantarum* A6.

Polyphenols, tannins, and phytates are known to reduce the availability of minerals such as iron and to bind to macromolecules such as proteins and carbohydrates, reducing their digestibility in foods [49]. Thus, by decreasing total phenolic compounds, tannins, and phytate content, the fermentation of the precooked Bambara groundnut flour, sorghum flour, and their mixture with *Lb. plantarum* leads to the improvement of their nutritional quality.

### 3.4. Physical, Functional, and Pasting Properties of Blended Flours

#### 3.4.1. Colour

The colour parameters *L*^∗^, *a*^∗^, and *b*^∗^ of treated samples are presented in Table 3. Colour parameters are important criteria for the acceptability of food products. A lower *L*^∗^ value indicates a darker flour, a positive *a*^∗^ value is associated with redness, and a positive *b*^∗^ value indicates yellow colour. The sorghum and Bambara groundnut blended flours have whitish colour and good clarity, with high values of *L*^∗^ (82.23 (SVP25), 80.45 (SVP50), and 81.96 (SVP75)). The *L*^∗^ values decreased with fermentation up to 79.06 for SVFP25, 77.60 for SVFP50, and 76.70 for SVFP75. The decrease of *L*^∗^ value after fermentation could be due to the breakage of cell cytoplasm with the liberation of some pigmentation or to the enzymatic browning. Siddiqi et al. [50] reported that the fermentation of dough of whey protein reduces *L*^∗^ value. The *a*^∗^ and *b*^∗^ values of the samples increased with fermentation but were not influenced by the mixture proportion.

#### 3.4.2. Particle Size Distribution

The effect of lactic fermentation and flour proportion on the particle size distribution of precooked flours is shown in Table 4. After fermentation, a significant reduction of the flour particle size was observed, from 205 *μ*m (SVP50) to 157 *μ*m (SVFP50) and from 212 *μ*m (SVP75) to 171 *μ*m (SVFP75). The size of the particles influenced the functional properties and the texture of the final product. The reduction of the particle size by fermentation can improve the smoothness of the gruel prepared with these flours. The fine particles of flour are important for fast hydration and cooking. Thus, these fermented flours used for the preparation of the gruel will require less time and energy for their hydration and cooking to that of unfermented flours. This result is similar to that of Oladeji [51], who showed that the particle size of unfermented maize was bigger than that of fermented samples. The high proportion of Bambara groundnut in the mixture also reduced the particle size of blended flours. This is because roasted Bambara groundnut has a smaller particle size (98.745 *μ*m) than precooked sorghum flour (196.397 *μ*m).

#### 3.4.3. Functional Properties

**Figure 3:**
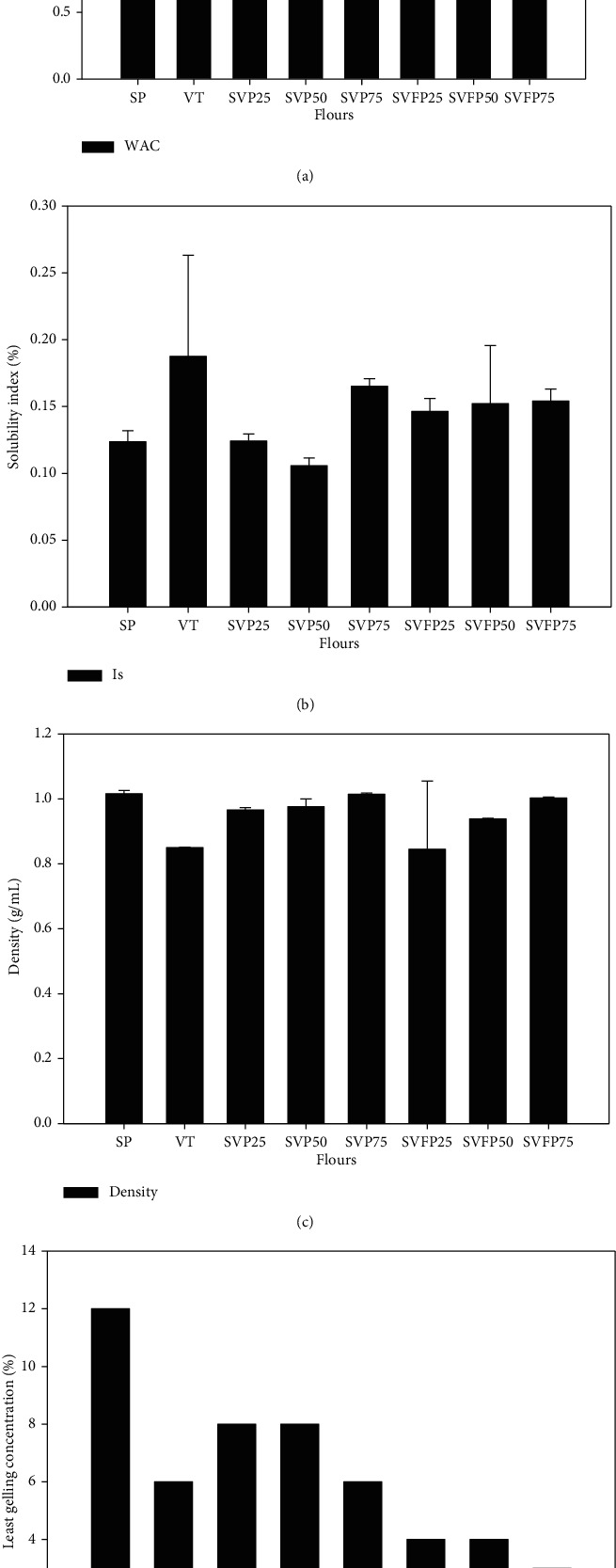
Water absorption capacity (a), solubility (b), density (c), and the least gelling concentration (d) of flours. SP: precooked dehulled sorghum flour; VT: roasted dehulled Bambara Flour; SVP25: blended flour containing 25% of precooked sorghum flour and 75% of roasted Bambara flour; SVP50: blended flour containing 50% of precooked sorghum flour and 50% of roasted Bambara flour; SVP75: blended flour containing 75% of precooked sorghum flour and 25% of roasted Bambara flour; SVFP25: fermented flour containing 25% of precooked sorghum flour and 75% roasted Bambara flour; SVFP50: fermented flour containing 50% precooked sorghum flour and 50% roasted Bambara flour; SVFP75: fermented flour containing 75% precooked sorghum flour and 25% roasted Bambara flour.

The functional properties of food ingredients or raw materials play an important role in the formulation of food products. These functional properties influence the sensory properties, hence, consumer acceptability of food products. They include, in the case of infant flours, water absorption capacity, solubility, density, and least gelling concentration. The water absorption capacity, solubility index, density, and the least gelling concentration of the samples are presented in Figure 3. The fermentation of the mixtures SVFP25, SVFP50, and SVFP75 reduced their water absorption capacity from 1.6, 1.71, and 1.80 g of water/g of flour to 1.55, 1.63, and 1.79, respectively. A similar observation has been reported by Ogodo et al. [52] on lactic acid bacteria fermented soybean flour. The reduction in water absorption capacity of an instant flour after fermentation is interesting as it can allow the preparation of gruels with low moisture content and high energetic density suitable for infant nutrition.

After the fermentation of the mixtures, there was a slight decrease in density of the samples with the highest value recorded with SVP75 (1.01 g/mL) and the lowest value with SVFP25 (0.84 g/mL). This reduction would probably be due to the breakage of the intermolecular link making the granules more porous and less dense. Jude-Ojei et al. [53] reported similar results on maize “Ogi” supplemented with fermented moringa seeds. Since the bulk density is highest in sorghum flour than in Bambara flour, it affects the bulk density of the mixture. In fact, when the sorghum precooked flour is added to the mixture, more is the bulk density of mixture.

The least gelling concentration (LGC) is shown in Figure 3(d). The incorporation of Bambara groundnut flour in the mixture decreased the LGC from 12% in precooked sorghum to 6% for the mixture containing 75% of Bambara groundnut flour. Fermentation also decreased the LGC up to 3% for SVFP75. The decrease of the LGC of fermented flours could be explained by the hydrolysis of starch by the amylases of *Lb. plantarum* A6 [54]. This reduces the ability of starch molecules to form networks that trap water molecules and thus allow the preparation of fluid gruel with high dry matter. Indeed, the fermentation of blended precooked sorghum and Bambara groundnut flours increased their solubility index from 0.10% for SVP50 and 0.12% for SVP25 to 0.15% for SVFP25 and 0.17% SVFP75. Ogodo et al. [52] and Rahma et al. [54] also reported a similar result after fermentation of soybean flour and *Treculia africana*, respectively.

#### 3.4.4. Pasting Properties

**Figure 4:**
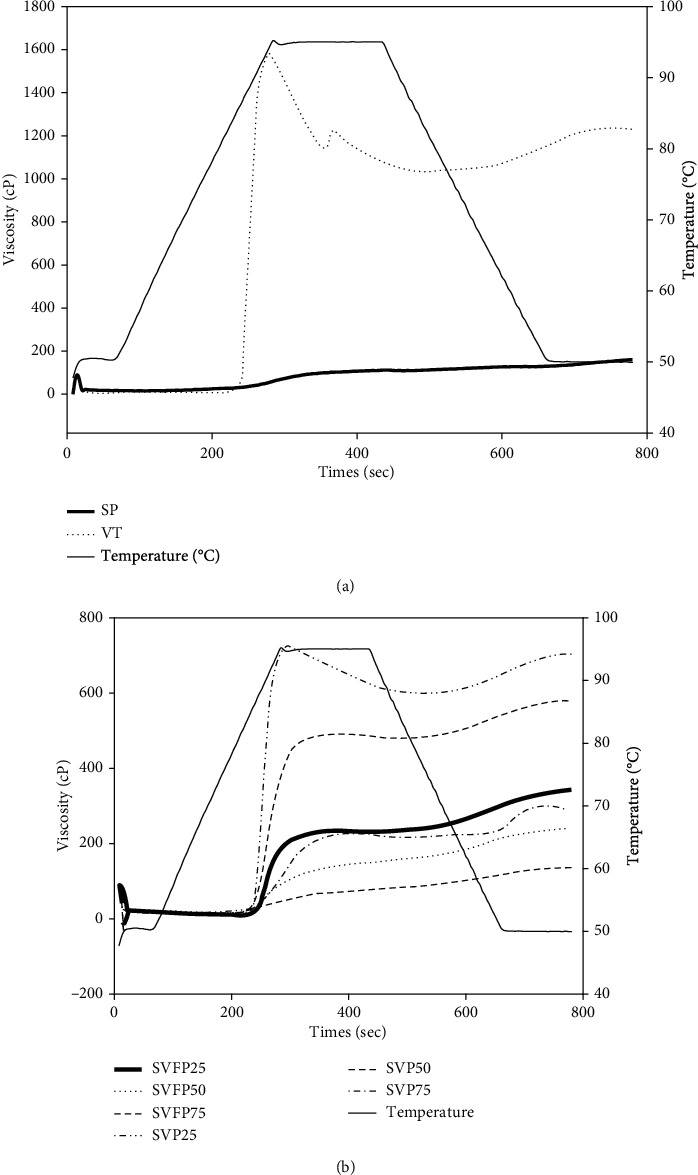
Pasting properties of precooked sorghum and Bambara groundnut flour (a) and blended precooked and fermented flour (b). SP: precooked dehulled sorghum flour; VT: roasted dehulled Bambara flour; SVP25: blended flour containing 25% of precooked sorghum flour and 75% of roasted Bambara flour; SVP50: blended flour containing 50% of precooked sorghum flour and 50% of roasted Bambara flour; SVP75: blended flour containing 75% of precooked sorghum flour and 25% of roasted Bambara flour; SVFP25: fermented flour containing 25% of precooked sorghum flour and 75% roasted Bambara flour; SVFP50: fermented flour containing 50% precooked sorghum flour and 50% roasted Bambara flour; SVFP75: fermented flour containing 75% precooked sorghum flour and 25% roasted Bambara flour.

Pasting properties showing the behavior of flours upon heating and cooling of fermented and unfermented samples are presented in Figure 4 and Table 5.

The pasting temperature (*P*_temp_) of unfermented samples (SVP25, SVP50, and SVP75) (Table 4) were 85.75, 85.52, and 85.15°C and are higher than the pasting temperature of fermented samples (SVFP25, SVFP50, and SVFP75) (85.03, 84.93, and 83.51°C). Fermentation reduced the minimum cooking temperature of blended flours, and the fermented flour can swell more quickly than unfermented flour. Peak viscosity (Pv) denotes the water-holding capacity of flours, and the final viscosity (Fv) indicates the ability of the sample to form a viscous paste after cooking and cooling. The peak viscosity of unfermented flours was 726 cP, 491 cP, and 226 cP for SVP25, SVP50, and SVP75, respectively. These Pv values decreased after fermentation up to 234 cP, 134 cP, and 69 cP for SVFP25, SVFP50, and SVFP75, respectively. On the other hand, the final viscosity of unfermented flours was 726 cP, 491 cP, and 226 cP for SVP25, SVP50, and SVP75, respectively. 704 cP, 579 cP, and 290 cP for SVP25, SVP50, and SVP75, respectively. Similarly to Pv values, the Fv values decreased after fermentation up to 342 cP, 240 cP, 136 cP for SVP25, SVP50, and SVP75, respectively. This can be due to the unfolding of starch during precooking with the subsequent increase of its accessibility to the hydrolytic enzyme, bacterial amylase during fermentation. Similarly, Nago [55] showed a decrease in viscosity after the fermentation of “ogui.” Nguyen et al. [8] reported that a combination of cooking and lactic fermentation considerably reduced the viscosity of the gruels prepared with rice and soybean blended flour. The peak viscosity also increased with a high level of Bambara groundnut flour in the mixture. This is due to the high protein contents in Bambara groundnut flour, which can gel during the cooking and contribute to increase the viscosity of the flour.

Hot paste viscosity (HPV) or holding strength indicates a shear-thinning property of the flour sample. It can be seen in Table 3 that roasted Bambara groundnut has a higher HPV (1038 cP) than precooked sorghum (109 cP) and the blended flours. This can be due to the resistance of its starch to shear stress and high temperature or the contribution of other components of its flours, mainly proteins, to the formation of heat-stable gel. When Bambara groundnut flour was mixed with sorghum flour, the HPV decreased significantly up to 598 cP, 479 cP, and 217 cP for SVP25, SVP50, and SVP75 samples, respectively. The fermentation also reduced significantly HPV; thus, these fermented flour can be used to prepare gruels with low viscosity and high caloric value. This is in line with the report of Akintayo et al. [56] suggesting that modification of processing techniques may help to reduce the viscosity of a gruel and that the lower the viscosity of a gruel, the better its suitability for infants. The breakdown (BD) of roasted Bambara groundnut flour (547 cP) was about two hundred times higher than precooked sorghum flour (3 cP). These may be due to the total gelatinization of sorghum starch during precooking. For the different mixtures, the BD decreased with the increase of the proportion of sorghum flour with 128 cP for 25% sorghum (SVP25), 12 cP for 50% (SVP50), and 9 cP for 75% (SVP75). Lactic acid fermentation significantly decreased the breakdown of blended flours from 128 cP (SVP25), 12 cP (SVP50), and 9 cP (SVP75) to 2 cP (SVFP25), 1 cP (SVFP50), and 1 cP (SVFP75), respectively. However, fermentation did not significantly affect the setback (SB) of the samples; thus, the fermentation did not affect the viscosity of the gruel prepared with these flours after cooling.

## 4. Conclusion

To our knowledge, this study is the first that has established the effect of lactic fermentation on precooked sorghum and Bambara groundnut blended flours. It appears at the end of the study that lactic acid fermentation affected the chemical composition of the flours by improving protein content making it possible to increase the protein and energy intake in the nutrition of young children. On the other hand, when the flours are first precooked, fermentation further reduces the viscosity of the sorghum/Bambara groundnut flour mixtures; this will result in a fluid porridge but more dense in nutrients and energy necessary for the child's growth. The fermentation of precooked sorghum, Bambara groundnut flours, and their mixture using *Lb. plantarum* A6 also reduces antinutrients of complex proteins and minerals, making them more available for the infant's absorption. From the overall results, the fermented blended flour containing 25% precooked sorghum flour and 75% roasted Bambara flour (SVFP25) would seem to be a promising alternative as instant flour for young children's nutrition.

## Figures and Tables

**Table 1:** Proximate composition of precooked fermented and unfermented flours.

Samples	Dry matter (g/100 g DM)	Ash (g/100 g DM)	Lipids (g/100 g DM)	Proteins (g/100 g DM)	Total sugars (g/100 g DM)	Crude fiber (g/100 g DM)	Energy (kcal/100 g DM)
SP	91.23 ± 0.47^a^	2.59 ± 0.02^c^	3.55 ± 0.08^a^	12.10 ± 0.06^a^	70.28 ± 0.46^h^	1.70 ± 0.25^a^	368.43 ± 0.12^a^
VT	94.73 ± 0.23^e^	2.17 ± 0.12^b^	9.01 ± 0.72^f^	19.52 ± 0.30^g^	60.35 ± 0.53^a^	1.20 ± 0.29^a^	400.61 ± 4.72^e^
SVP 25	95.6 ± 0.20^f^	2.27 ± 0.21^b^	7.23 ± 0.54^d^	18.42 ± 0.24^f^	62.76 ± 0.39^b^	1.40 ± 0.52^c^	389.79 ± 3.87^d^
SVP 50	94.0 ± 0.34^d^	2.38 ± 0.06^b^	6.20 ± 0.05^c^	16.13 ± 0.17^d^	64.88 ± 0.27^e^	1.54 ± 0.26^c^	380.56 ± 0.60^c^
SVP 75	93.13 ± 0.23^c^	2.48 ± 0.22^b^	5.14 ± 0.04^b^	14.20 ± 0.11^b^	67.58 ± 0.23^f^	1.76 ± 0.33^c^	373.38 ± 0.90^b^
SVFP 25	94.53 ± 0.64^de^	1.86 ± 0.05^a^	8.15 ± 0.15^e^	19.98 ± 0.30^f^	60.66 ± 0.07^c^	2.09 ± 0.45^c^	395.95 ± 1.62^d^
SVFP 50	92.13 ± 0.23^b^	2.20 ± 0.27^b^	6.46 ± 0.07^c^	17.91 ± 0.25^e^	62.51 ± 0.32^d^	2.23 ± 0.35^c^	376.22 ± 2.64^b^
SVFP 75	94.0 ± 0.40^d^	2.91 ± 0.22^c^	5.47 ± 0.36^b^	15.88 ± 0.20^c^	64.37 ± 0.17^e^	2.52 ± 0.49^c^	370.23 ± 1.49^c^

In the same column, the values followed by the same superscript letters are not significantly different (*p* < 0.05). SP: precooked dehulled sorghum flour; VT: roasted dehulled Bambara flour; SVP25: blended flour containing 25% of precooked sorghum flour and 75% of roasted Bambara flour; SVP50: blended flour containing 50% of precooked sorghum flour and 50% of roasted Bambara flour; SVP75: blended flour containing 75% of precooked sorghum flour and 25% of roasted Bambara flour; SVFP25: fermented flour containing 25% of precooked sorghum flour and 75% roasted Bambara flour; SVFP50: fermented flour containing 50% precooked sorghum flour and 50% roasted Bambara flour; SVFP75: fermented flour containing 75% precooked sorghum flour and 25% roasted Bambara flour.

**Table 2:** Antinutrient composition of precooked fermented and unfermented flours.

Samples	Total phenolic compound (mg/100 g DM)	Tannins (mg/100 g DM)	Phytate (mg/100 g DM)
SP	34.01 ± 0.03^b^	28.67 ± 0.01^b^	50.67 ± 0.04^b^
VT	138.01 ± 0.09^f^	57.33 ± 0.03^f^	166.53 ± 0.013^f^
SVP 25	117.02 ± 0.06^e^	50.16 ± 0.02^e^	137.16 ± 0.010^e^
SVP 50	96.03 ± 0.03^d^	43.01 ± 0.02^d^	108.33 ± 0.08^d^
SVP 75	65.01 ± 0.01^c^	35.83 ± 0.01^c^	79.50 ± 0.06^c^
SVFP 25	29.05 ± 0.03^ab^	11.41 ± 0.01^a^	41.17 ± 0.03^ab^
SVFP 50	27.33 ± 0.01^ab^	11.15 ± 0.01^a^	35.01 ± 0.01^a^
SVFP 75	25.16 ± 0.027^a^	11.58 ± 0.01^a^	28.83 ± 0.01^a^

In the same column, the values followed by the same superscript letters are not significantly different (*p* < 0.05). SP: precooked dehulled sorghum flour; VT: roasted dehulled Bambara flour; SVP25: blended flour containing 25% of precooked sorghum flour and 75% of roasted Bambara flour; SVP50: blended flour containing 50% of precooked sorghum flour and 50% of roasted Bambara flour; SVP75: blended flour containing 75% of precooked sorghum flour and 25% of roasted Bambara flour; SVFP25: fermented flour containing 25% of precooked sorghum flour and 75% roasted Bambara flour; SVFP50: fermented flour containing 50% precooked sorghum flour and 50% roasted Bambara flour; SVFP75: fermented flour containing 75% precooked sorghum flour and 25% roasted Bambara flour.

**Table 3:** Colour of flours.

Samples	*L* ^∗^	*a* ^∗^	*b* ^∗^
SP	78.65 ± 0.98^ab^	4.68 ± 0.24^a^	14.15 ± 0.24^a^
VT	84.59 ± 5.21^c^	7.96 ± 4.01^bc^	22.85 ± 7.46^bc^
SVP 25	82.33 ± 0.33^bc^	9.20 ± 0.24^c^	25.71 ± 0.63^c^
SVP 50	80.45 ± 1.94^ab^	8.23 ± 0.60^bc^	23.69 ± 1.12^bc^
SVP 75	81.96 ± 0.96^bc^	6.16 ± 0.18^ab^	19.77 ± 0.22^b^
SVFP 25	79.06 ± 0.54^ab^	9.22 ± 0.07^c^	23.07 ± 0.21^bc^
SVFP 50	77.60 ± 0.57^a^	9.03 ± 0.07^c^	23.23 ± 0.31^bc^
SVFP 75	76.70 ± 1.08^a^	8.41 ± 0.24^bc^	20.73 ± 0.28^bc^

In the same column, the values followed by the same letters in exponent are not significantly different (*p* < 0.05). SP: precooked dehulled sorghum flour; VT: roasted dehulled Bambara flour; SVP25: blended flour containing 25% of precooked sorghum flour and 75% of roasted Bambara flour; SVP50: blended flour containing 50% of precooked sorghum flour and 50% of roasted Bambara flour; SVP75: blended flour containing 75% of precooked sorghum flour and 25% of roasted Bambara flour; SVFP25: fermented flour containing 25% of precooked sorghum flour and 75% roasted Bambara flour; SVFP50: fermented flour containing 50% precooked sorghum flour and 50% roasted Bambara flour; SVFP75: fermented flour containing 75% precooked sorghum flour and 25% roasted Bambara flour.

**Table 4:** Particle size distribution parameters (*μ*m).

Samples	d10	d50	d90	Means
SP	14.977	130.506	489.271	196.397
VT	18.465	62.814	235.002	98.745
SVP 25	19.882	82.935	343.394	138.114
SVP 50	22.461	117.385	342.531	165.363
SVP 75	25.795	170.006	442.437	205.122
SVFP 25	50.849	172.886	437.782	212.943
SVFP 50	18.588	111.416	368.797	157.785
SVFP 75	21.404	145.621	364.546	171.822

SP: precooked dehulled sorghum flour; VT: roasted dehulled Bambara flour; SVP25: blended flour containing 25% of precooked sorghum flour and 75% of roasted Bambara flour; SVP50: blended flour containing 50% of precooked sorghum flour and 50% of roasted Bambara flour; SVP75: blended flour containing 75% of precooked sorghum flour and 25% of roasted Bambara flour; SVFP25: fermented flour containing 25% of precooked sorghum flour and 75% roasted Bambara flour; SVFP50: fermented flour containing 50% precooked sorghum flour and 50% roasted Bambara flour; SVFP75: fermented flour containing 75% precooked sorghum flour and 25% roasted Bambara flour.

**Table 5:** Visco-analysis flours pasting profiles of fermented and unfermented flours.

Samples	RVA viscosity, cP					Pasting temperature, °C (*P*_temp_)
	Peak (Pv)	Holding (HPV)	Breakdown (BD)	Final (FV)	Setback (SB)	
SP	112	109	3	158	49	76.20
VT	1585	1038	547	1235	197	86.79
SVP25	726	598	128	704	106	85.75
SVP50	491	479	12	579	100	85.52
SVP75	226	217	9	290	73	85.15
SVFP25	234	232	2	342	110	85.03
SVFP50	134	133	1	240	107	84.93
SVFP75	69	68	1	136	68	83.51

SP: precooked dehulled sorghum flour; VT: roasted dehulled Bambara flour; SVP25: blended flour containing 25% of precooked sorghum flour and 75% of roasted Bambara flour; SVP50: blended flour containing 50% of precooked sorghum flour and 50% of roasted Bambara flour; SVP75: blended flour containing 75% of precooked sorghum flour and 25% of roasted Bambara flour; SVFP25: fermented flour containing 25% of precooked sorghum flour and 75% roasted Bambara flour; SVFP50: fermented flour containing 50% precooked sorghum flour and 50% roasted Bambara flour; SVFP75: fermented flour containing 75% precooked sorghum flour and 25% roasted Bambara flour.

## Data Availability

Data are available on request. To request the data, please contact Pr. Leopold NGOUNE TATSADJIEU, University Institute of Technology, Department of Food Engineering and Quality Control, University of Ngaoundere, P.O Box 454 Ngaoundere, Cameroon; Cameroon tatsadjieu@yahoo.fr; Tel.: +237 699523727.
